# Candidate Biomarkers and Molecular Mechanism Investigation for Glioblastoma Multiforme Utilizing WGCNA

**DOI:** 10.1155/2018/4246703

**Published:** 2018-09-26

**Authors:** Qi Yang, Rui Wang, Bo Wei, Chuangang Peng, Le Wang, Guozhang Hu, Daliang Kong, Chao Du

**Affiliations:** ^1^Department of Gynecology and Obstetrics, China-Japan Union Hospital of Jilin University, Changchun, Jilin 130033, China; ^2^Department of Radiology, China-Japan Union Hospital of Jilin University, Changchun, Jilin 130033, China; ^3^Departments of Neurosurgery, China-Japan Union Hospital of Jilin University, Changchun, Jilin 130033, China; ^4^Orthopaedic Medical Center, The 2nd Hospital of Jilin University, Changchun, Jilin 130041, China; ^5^Department of Ophthalmology, The First Hospital of Jilin University, Changchun, Jilin 130041, China; ^6^Department of Emergency Medicine, China-Japan Union Hospital of Jilin University, Changchun, Jilin 130033, China; ^7^Departments of Orthopaedics, China-Japan Union Hospital of Jilin University, Changchun, Jilin 130033, China

## Abstract

To reveal the potential molecular mechanism of glioblastoma multiforme (GBM) and provide the candidate biomarkers for GBM gene therapy. Microarray dataset GSE50161 was obtained from GEO database. The differentially expressed genes (DEGs) were identified between GBM samples and control samples, followed by the module partition analysis based on WGCNA. Then, the pathway and functional enrichment analyses of DEGs were performed. The hub genes were further investigated, followed by the survival analysis and data validation. A total of 1913 DEGs were investigated between two groups, followed by analysis of 5 modules using WGCNA. These DEGs were mainly enriched in functions like inflammatory response. The hub genes including upregulated N-Myc and STAT Interactor (NMI), Capping Actin Protein-Gelsolin Like (CAPG), and Proteasome Subunit Beta 8 (PSMB8) were revealed as potential liquid biopsy molecules for GBM diagnose. Moreover, Nucleolar and Spindle Associated Protein 1 (NUSAP1) and G Protein-Coupled Receptor 65 (GPR65) were outstanding genes in survival analysis. Our results suggested that CPNE6, HAPLN2, CMTM3, NMI, CAPG, and PSMB8 might be used as potential molecules for liquid biopsy of GBM. NUSAP1 and GPR65 might be novel prognostic targets for GBM gene therapy. Furthermore, the upregulated NMI might play an important role in GBM progression via inflammatory response.

## 1. Introduction

Glioblastoma multiforme (GBM) is the most aggressive cancer that represent 15% of all brain tumors [1]. The survival rate for GBM patients is less than 15 months [2]. Although surgery is commonly used for the treatment of GBM [3], the cancer usually recurs due to lack of effective prevention method for this disease [4].

Understanding the mechanisms of glioblastoma at the molecular and structural level is valuable for clinical treatment [5]. Bioinformatics can be effectively used to analyze GBM microarray data to provide theoretical reference for further exploration of tumorigenesis mechanism and help search for potential target genes [6]. Based on bioinformatics study, some differentially expressed genes (DEGs) such as Transforming Growth Factor Beta Induced (TGFBI) and SRY-Box 4 (SOX4) were explored as the potential therapy targets for GBM [7]. Coexpression analysis has emerged as a powerful technique for obtaining novel insights into complex mechanisms and multigene analysis of large-scale data sets, especially for identifying functional modules. As an approach of bioinformatics study, weighted gene coexpression network analysis (WGCNA) is commonly used for revealing the correlation between genes in different samples [8]. Previous WGCNA shows the epigenetic events in GBM development and prognosis based on The Cancer Genome Atlas (TCGA) database [9]. Thus, WGCNA can be used to predict genes associated with cancer development [10]. In pervious study, Griesinger et al. indicated that the different pediatric brain tumor types (including GBM) exhibited distinct immunophenotypes, implying that specific immunotherapeutic approaches may be most effective for each tumor type [11]. However, candidate biomarkers for the clinical gene therapy of GBM were still unclear.

In current study, GBM gene expression data deposited by Griesinger et al. [11] was downloaded and reanalyzed by WGCNA. The DEGs between GBM samples and control samples were investigated, followed by the functional and pathway enrichment analysis. Then, TCGA survival analysis and data validation as well as literature verification were performed to confirm the effect of biomarker for GBM. We hoped to reveal the potential molecular mechanism of GBM and provide the candidate biomarkers for GBM gene therapy.

## 2. Material and Methods

### 2.1. Microarray Data

The gene expression profile of GSE50161 [11] was downloaded from Gene Expression Omnibus (GEO) database [12]. A total of 47 tissue samples including 34 surgical brain tissue samples (GBM group) obtained from patients who were diagnosed with GBM and 13 normal brain samples (control group) obtained from pediatric epilepsy patients at the time of surgical intervention were used for the follow-up analysis.

### 2.2. Data Preprocessing and DEG Analysis

There were totally 17049 probes in the present dataset. The knn function in the impute package (version: 1.48.0) [13] was used to impute missing value. Normalization was performed using Limma Linear Models for Microarray Data (limma, version: 3.30.13) package [14]. The median value was considered as the gene expression value when different probes linked to the same gene (16812 genes). The eBayes analysis [15] was used to analyze the DEGs between GBM group and control group. P-value <0.05 and |log-fold change (LFC)| >2 were selected as the thresholds for DEGs screening.

### 2.3. WGCNA Analysis

The coexpression network analysis was performed using WGCNA (version:1.61) [8]. First, the soft threshold for network construction was selected. The soft threshold makes the adjacency matrix to be the continuous value between 0 and 1, so that the constructed network conforms to the power-law distribution and is closer to the real biological network state. Second, the scale-free network was constructed using blockwiseModules function, followed by the module partition analysis to identify gene coexpression modules, which could group genes with similar patterns of expression. The modules were defined by cutting the clustering tree into branches using a dynamic tree cutting algorithm and assigned to different colors for visualization [16]. Then, the module eigengene (ME) of each module was calculated. ME represents the expression level for each module. Then, the correlation between ME and clinical trait in each module was calculated. Finally, the gene significance (GS) of gene in the module, which represented the correlation between gene and sample, was further calculated.

### 2.4. Function and the Pathway Enrichment Analysis

The DAVID 6.8 (https://david.ncifcrf.gov) [17] software was used for the GO-biological function (GO-BP) [18] and KEGG pathway analysis [19] of genes in each module. P  _false  discovery  rate  (FDR)_ <0.05 was selected as the threshold for the identification of significant GO-BP terms and pathways.

### 2.5. The Hub Gene Investigation

The network topological index is defined as the number of links incident upon a node [20]. The key node (hub gene) was determined by high intramodule connectivity of genes by summing the connection strengths with other module genes. In this study, the intramodular connectivity of genes was identified by WGCNA [8]. According to the intramodule connectivity, the top 20 hub genes in modules were visualized using Cytoscape (version 3.5.1) software [21].

### 2.6. Survival Analysis

To reveal the prognostic value of hub genes on GBM patients, the survival analysis was performed. The expression value and clinical data of GBM were downloaded from the TCGA [22] database. A total of 148 samples were included. The samples in the data were divided into high expression group (up group) and low expression group (down group) according to the median value of each hub protein. The survival package (version: 2.41-3) in R software was used for the current analysis. Then, the survival rate estimation and statistical significance were performed using Kaplan-Meier (KM) method [23] and log-rank test [24], respectively. P < 0.05 was considered statistically significant.

### 2.7. Data Validation

In order to verify the expression of hub genes in serum samples in other dataset, the microarray data of GSE24084 [25] (platform: GPL8755 PF-Agilent-014850 Whole Human Genome Microarray 4x44K), which included 16 serum samples (9 cancer samples and 7 normal samples), was obtained from GEO database. Using ROCR package [26] in R software (version: 1.0-7), the area under curve (AUC) from the receiver operating characteristic (ROC) curve study was performed on the expression data of each hub gene. In the present study, AUC >0.5 represented upregulated genes, while AUC <0.5 represented downregulated genes. Larger |AUC-0.5| value of a gene indicated that this genes can well distinguish GBM from the control samples [27]. Based on the AUC values, the diagnose effect of hub genes (as liquid biopsy molecules) in each module was further investigated.

## 3. Results

### 3.1. DEGs between GBM Group and Control Group

**Figure 1:**
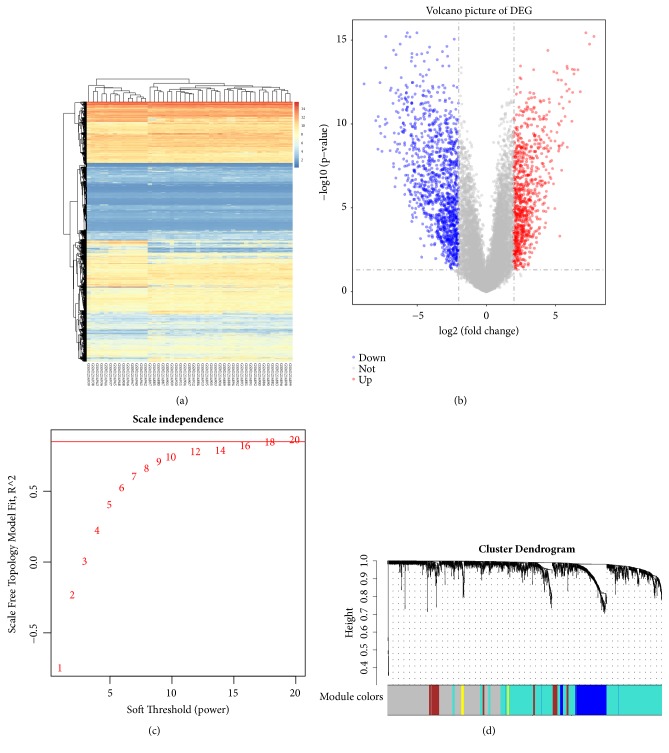
The heat map, volcano plot, and weighted gene coexpression network analysis (WGCNA) of differentially expressed genes (DEGs) between glioblastoma multiforme group and control group. (a) The heat map for DEGs. (b) The volcano plot for DEGs. Grey dots represent genes which are not differentially expressed, red dots represent the upregulated genes, and the blue dots represent the downregulated genes. (c) Determination of the soft threshold in the WGCNA algorithm. The approximate scale-free fit index can be attained at the soft-thresholding power of 18. (d) Clustering dendrograms showing 5 modules that contain a group of highly connected genes. Each designated color represents a certain gene module.

With P_FDR_< 0.05 and |LFC|> 2, a total of 1913 DEGs including 776 upregulated DEGs and 1137 downregulated DEGs were identified between GBM group and control group. The heat map and volcano plot are shown in Figures 1(a) and 1(b), respectively. The results of heat map showed that these DEGs could be used to well distinguish the GBM from the control samples.

### 3.2. WGCNA Analysis

The WGCNA analysis was performed on 1913 DEGs. The soft threshold for network construction was selected as 18 (Figure 1(c)) [28]. Meanwhile, the fitting degree of scale-free topological model was 0.85. Thus, this network conformed to the power-law distribution and was closer to the real biological network state.

**Figure 2:**
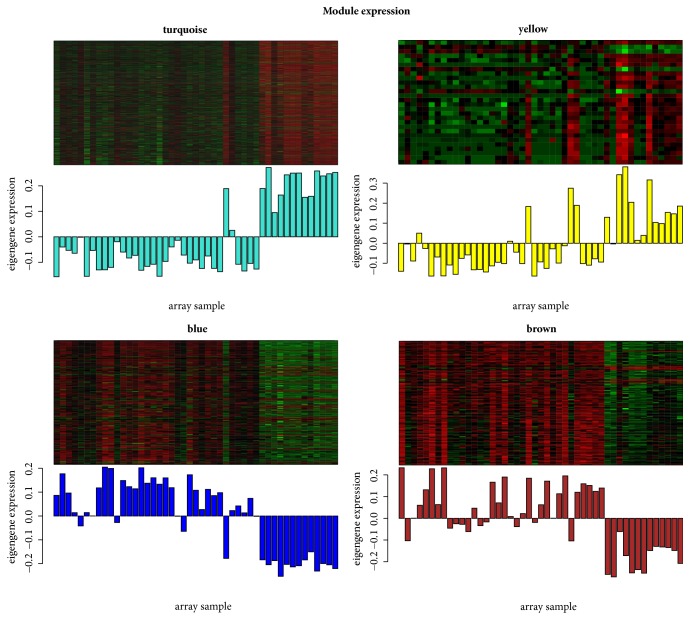
The module expression pattern. The heat map represents the expression of genes where each row represents a gene and each column represents a sample. The red color in heat map represents upregulated genes while the green color represents the downregulated gene. The bar charts represents the eigengene profiles of four WGCNA modules; the color of the bar chart represented the color of related module.

**Figure 3:**
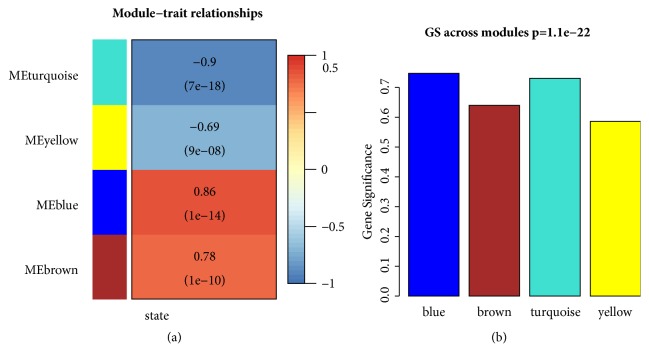
Relationships of module eignegenes and the samples. (a) The module-trait relationships. Module names are displayed on the left. The number in the first row of the square was the correlation coefficients to the glioblastoma multiforme (GBM) group shown at the top of each row with the p values printed below the correlations in parentheses. The rows are colored based on the correlation of the module to the GBM group: red for positive correlation and blue for negative correlation. (b) The average gene significance (GS) of all genes (i.e., module significance, MS) of each module. Modules with greater MS values were considered to have more connection with the disease.

A total of 5 modules (Figure 1(d)) including turquoise (867 DEGs), grey (643 DEGs), blue (238 DEGs), brown (137 DEGs), and yellow (28 DEGs) were obtained in current study. The DEGs in grey were not included in any module, so the subsequent analysis was no long performed on grey. ME was in accordance with the expression pattern of DEGs in each module. The turquoise module and yellow module were downregulated, while blue module and brown module were upregulated. Furthermore, the turquoise module (correlation index: -0.9, P =7.0E-18) and yellow module (correlation index: -0.69, P =9.0E-8) were negatively correlated with the disease. Meanwhile, blue module (correlation index: 0.86, P =1.0E-14) and brown module (correlation index: -0.78, P =1.0E-10) were positively correlated with the disease (Figure 2). The GS value for blue module, turquoise module, brown module, and yellow module were 0.75, 0.73, 0.64, and 0.59, respectively, which indicated a close relationship with the disease (Figure 3).

### 3.3. Functional and Pathway Enrichment for DEGs

The top 3 of GO-BP and KEGG terms enriched by DEGs were showed in Table 1. The result showed that DEGs in turquoise module were mainly involved in the functions like chemical synaptic transmission (GO:0007268, P =6.65E-29) and pathways such as retrograde endocannabinoid signaling (hsa04723, P =1.76E-10, genes). The DEGs in the blue module were mainly associated with functions like cell division (GO:0051301, P =9.80E-40) and pathways like cell cycle (hsa04110, P =8.98E-15). Meanwhile, the DEGs in brown module were mainly involved in functions like inflammatory response (GO:0006954, P =2.05E-04) and pathways like Pertussis (hsa05133, P =5.25E-02). The enrichment result in yellow module was not significant because the number of genes in assembled in this module was less.

### 3.4. Hub Genes in Modules

**Figure 4:**
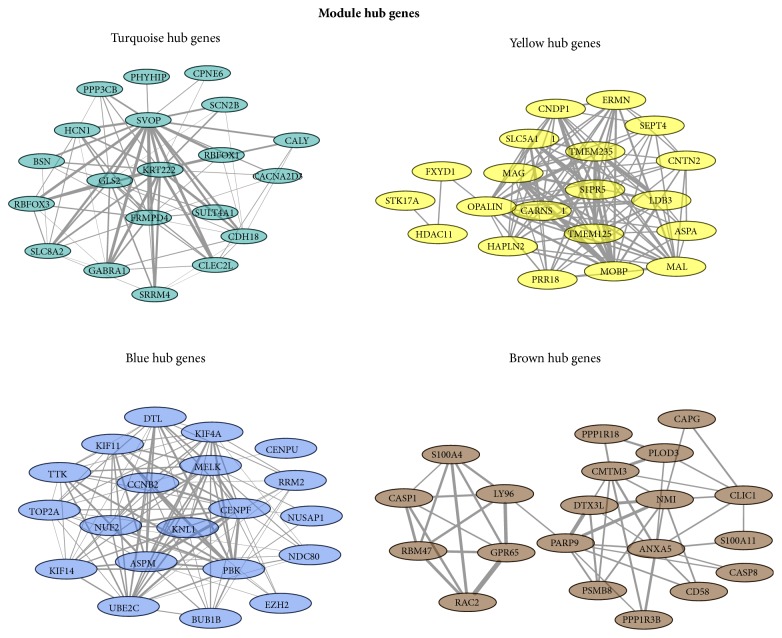
The visualization of modules with hub genes. The different colors represented the different modules. The thicker line represents higher connection strengths.

According to the network topological index, a total of 80 hub genes were investigated from 4 modules. The detailed information was showed in Figure 4.

### 3.5. Survival Analysis and Data Validation

**Figure 5:**
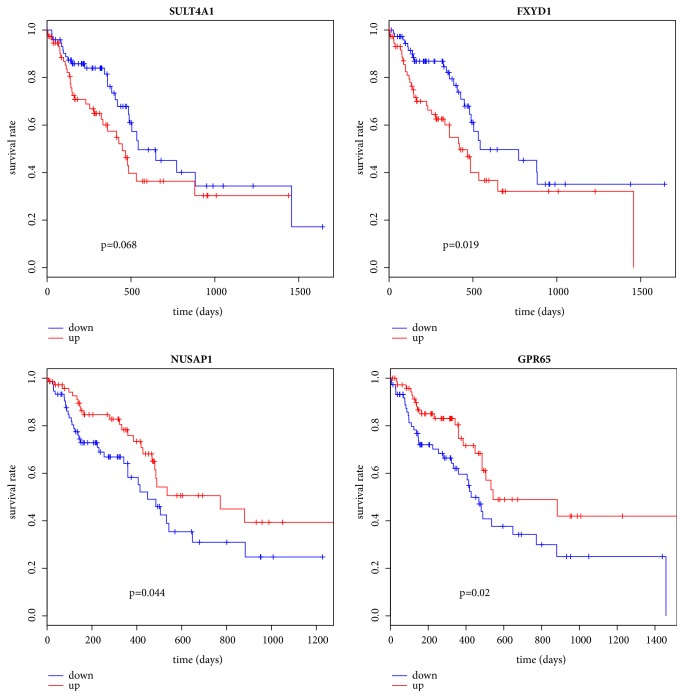
**Survival curve for testing hub genes in the modules in TCGA data. **FXYD1, NUSAP1, GPR65, and GPR65 were genes with the minimal P value in in yellow module, blue module, and brown module and turquoise module, respectively.

To validate our initial finding of hub genes, we examined an independent TCGA dataset. Based on the expression and clinical data of 148 GBM samples from TCGA database, the survival analysis was performed on totally 80 hub genes. The KM curve for gene with the minimal P value in each module was showed in Figure 5. Three genes including FXYD Domain Containing Ion Transport Regulator 1 (FXYD1, P =1.92E-02), Nucleolar and Spindle Associated Protein 1 (NUSAP1, P =4.44E-02), and G Protein-Coupled Receptor 65 (GPR65, P =2.01E-02) were outstanding in the current survival analysis (Figure 5), which had significant association with prognosis.

**Figure 6:**
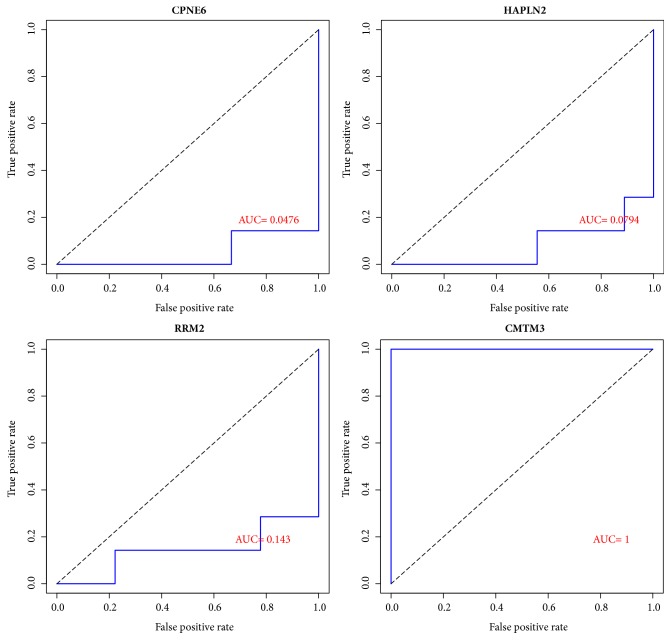
The receiver operating characteristic (ROC) curve of hub genes in GSE24084 dataset. CPNE6, HAPLN2, RRM2, and CMTM3 were four genes with the largest |AUC-0.5|.

Moreover, to explore the expression pattern of hub genes in serum samples from GBM patients, the validation data GSE24084 was downloaded from the GEO database, followed by ROC curve analysis using ROCR software. The result showed that Copine 6 (CPNE6), Hyaluronan and Proteoglycan Link Protein 2 (HAPLN2), Ribonucleotide Reductase Regulatory Subunit M2 (RRM2), and CKLF Like MARVEL Transmembrane Domain Containing 3 (CMTM3) were four outstanding genes with the largest |AUC-0.5| in each module, respectively (Figure 6). Furthermore, the other genes including N-Myc and STAT Interactor (NMI), Capping Actin Protein-Gelsolin Like (CAPG), Proteasome Subunit Beta 8 (PSMB8), CKLF Like MARVEL Transmembrane Domain Containing 3 (CMTM3), Sodium Voltage-Gated Channel Beta Subunit 2 (SCN2B), Copine 6 (CPNE6), and Hyaluronan and Proteoglycan Link Protein 2 (HAPLN2) were genes with |AUC-0.5| > 0.4.

## 4. Discussion

GBM is a kind of brain tumor in adults [29]. Targeted gene therapy is a strategy for GBM [30]. However, the effective candidate biomarkers for the gene therapy of GBM was still unclear. A gene expression data of GBM was analyzed by WGCNA in this study. The results showed that a total of 1913 DEGs were investigated between GBM samples and control samples, followed by 5 modules explored. These DEGs were mainly enriched in functions like inflammatory response and pathways including cell cycle. The hub genes including CPNE6, HAPLN2, CMTM3, NMI, CAPG, and PSMB8 were revealed as potential liquid biopsy molecules for GBM diagnose. Moreover, FXYD1, NUSAP1, and GPR65 were three outstanding genes in survival analysis.

Evidence demonstrated that CPNE6 terminated their expression in glioblastomas [31]. Sim and colleagues found that HAPLN2, also known as BRAL1, was virtually absent in malignant gliomas [32]. A previous study had reported that CMTM3 promoted cell invasion of glioblastoma and was significantly correlated with shorter overall survival [33]. NMI, is a protein that play critical roles in tumor growth, progression, and metastasis [34]. Previous study indicates that NMI polymorphisms are closely related to the genetic susceptibility of glioma in Chinese Han population [35]. CAPG is a ubiquitous gelsolin-family actin-modulating protein involved in the control of cell migration via immune and inflammatory pathways [36]. Yun et al. showed that the downregulation of CAPG significantly inhibited GBM cell proliferation by blocking the cell cycle in G1/S transition [37]. PSMB8, a protein contributes to the complete assembly of 20S proteasome complex, regulates glioma cell migration, proliferation, and apoptosis [38]. Previous study shows that autoinflammatory disorder caused by PSMB8 mutation leads to the proteasome assembly defection [39]. In the present study, the |AUC-0.5| value for hub genes like CPNE6 (downregulated), HAPLN2 (downregulated), CMTM3 (upregulated), NMI (upregulated), CAPG (upregulated), and PSMB8 (upregulated) was all larger than 0.4. Based on the study of Long* et al.* [27], we suggested that hub genes like CPNE6, HAPLN2, CMTM3, NMI, CAPG, and PSMB8 might be used as potential molecules for liquid biopsy of GBM. Furthermore the inflammatory pathway has previously been linked to chemotherapy resistance in glioma tumors [40]. Actually, the suppression role in inflammation response was closely related with gliomas development and progression [41]. In this study, the function analysis in this study showed that inflammatory response was one of the most outstanding functions enriched by DEGs including upregulated NMI. Thus, we speculated that the upregulated NMI might play an important role in GBM progression via inflammatory response.

Identification of hub genes or regulatory factors is the first step for GBM gene therapy [42]. Previous study shows that NUSAP1 expression is correlated not only with glioma grade but also with prognosis of glioma patients [43]. Knockdown of NUSAP1 expressing suppress glioma cells growth and promote glioma cell apoptosis [44]. Despite NUSAP1, GPR65 proved to promote tumor growth in cancer [45]. Ail et al. indicated that GPR65 may take part in a mechanism that is for photoreceptors survival in the degenerating retina [46]. However, whether GPR65 contribute to the process of glioma or BGM is still unclear. In the present study, NUSAP1 and GPR65 were not only DEGs between GBM samples and control samples, but also two most outstanding hub genes in survival analysis. Thus, we speculated that NUSAP1 and GPR65 might be novel prognostic targets for GBM gene therapy. However, there were some limitations in this study such as small sample size and lack of verification test; thus, a large sample size with a wide verification analysis is needed in the further investigation.

## 5. Conclusions

In conclusion, CPNE6, HAPLN2, CMTM3, NMI, CAPG, and PSMB8 might be used as potential molecules for liquid biopsy of GBM. NUSAP1 and GPR65 might be novel prognostic targets for GBM gene therapy. Furthermore, the upregulated NMI might play an important role in GBM progression via inflammatory response.

## Figures and Tables

**Table 1:** The results for GO-BP function and KEGG pathway enrichment analysis (top 3 in each module are listed).

**Module**	**GO-BP terms**	***P***_**FDR**_	**KEGG terms**	**P**_**FDR**_
Turquoise	GO:0007268~chemical synaptic transmission	6.65E-29	hsa04723: Retrograde endocannabinoid signaling	2.49E-14
	GO:0007269~neurotransmitter secretion	7.27E-15	hsa04727: GABAergic synapse	1.76E-10
	GO:0014047~glutamate secretion	7.47E-12	hsa05032: Morphine addiction	9.20E-10
Blue	GO:0051301~cell division	9.80E-40	hsa04110: Cell cycle	8.98E-15
	GO:0007067~mitotic nuclear division	8.74E-27	hsa03030: DNA replication	7.01E-05
	GO:0007062~sister chromatid cohesion	5.35E-22	hsa04114: Oocyte meiosis	1.74E-03
Brown	GO:0006954~inflammatory response	2.05E-04	hsa05133: Pertussis	5.25E-02
	GO:0030198~extracellular matrix organization	3.03E-03	hsa04064: NF-kappa B signaling pathway	1.21E-01
	GO:0051607~defense response to virus	4.84E-02	hsa04610: Complement and coagulation cascades	3.76E-01
Yellow	GO:0007417~central nervous system development	1.30E+01	hsa00340: Histidine metabolism	4.21E-01
	GO:0006915~apoptotic process	3.94E+01	hsa00410: beta-Alanine metabolism	3.06E+01
	GO:0055085~transmembrane transport	4.13E+01	hsa00330: Arginine and proline metabolism	4.45E+01

Notes: GO, gene-ontology; BP, biological process; KEGG, Kyoto Encyclopedia of Genes and Genomes; FDR, false discovery rate.

## Data Availability

The data used to support the findings of this study are available from the corresponding author upon request.

## References

[1] Ostrom Q. T., Gittleman H., Farah P. (2013). CBTRUS statistical report: primary brain and central nervous system tumors diagnosed in the United States in 2006–2010. *Neuro-Oncology*.

[2] Young R. M., Jamshidi A., Davis G., etal. (2015). Current trends in the surgical management and treatment of adult glioblastoma. *Ann Transl Med*.

[3] Nam J. Y., De Groot J. F. (2017). Treatment of glioblastoma. *Journal of Oncology Practice*.

[4] Gallego O. (2015). Nonsurgical treatment of recurrent glioblastoma. *Current Oncology*.

[5] Bo L. J., Wei B., Li Z. H. (2015). Bioinformatics analysis of miRNA expression profile between primary and recurrent glioblastoma. *European Review for Medical Pharmacological Sciences*.

[6] Guo Q., Zhang M., Hu G. (2018). Bioinformatics Analysis of Differentially Expressed Genes in Glioblastoma. *Acta Medicinae Universitatis Scientiae Et Technologiae Huazhong*.

[7] Lin B., Madan A., Fang X., Yoon J., Foltz G. (2014). Abstract 2224: Next-generation sequencing and bioinformatics analysis identified up-regulation of TGFBI and SOX4 in human glioblastoma. *Cancer Research*.

[8] Langfelder P., Horvath S. (2008). WGCNA: an R package for weighted correlation network analysis. *BMC Bioinformatics*.

[9] Xiang Y., Zhang C.-Q., Huang K. (2012). Predicting glioblastoma prognosis networks using weighted gene co-expression network analysis on TCGA data.. *BMC Bioinformatics*.

[10] Zhai X., Xue Q., Liu Q., Guo Y., Chen Z. (2017). Colon cancer recurrence-associated genes revealed by WGCNA co-expression network analysis. *Molecular Medicine Reports*.

[11] Griesinger A. M., Birks D. K., Donson A. M. (2013). Characterization of distinct immunophenotypes across pediatric brain tumor types. *The Journal of Immunology*.

[12] Barrett T., Troup D. B., Wilhite S. E. (2007). NCBI GEO: mining tens of millions of expression profiles—database and tools update. *Nucleic Acids Research*.

[13] Hastie T., Tibshirani R., Narasimhan B. (2011). Impute: Imputation for microarray data. *Oral History Review*.

[14] Gentleman R. C., Carey V., Hüber W., Irizarry R., Dudoit R. (2005). *Bioinformatics and Computational Biology Solutions Using R and Bioconductor*.

[15] Yang D., Parrish R. S., Brock G. N. (2014). Empirical evaluation of consistency and accuracy of methods to detect differentially expressed genes based on microarray data. *Computers in Biology and Medicine*.

[16] Langfelder P., Zhang B., Horvath S. (2008). Defining clusters from a hierarchical cluster tree: the dynamic tree cut package for R. *Bioinformatics*.

[17] Bozdech Z., Zhu J., Joachimiak M. P., Cohen F. E., Pulliam B., DeRisi J. L. (2003). Expression profiling of the schizont and trophozoite stages of *Plasmodium falciparum* with a long-oligonucleotide microarray. *Genome Biology*.

[18] Ashburner M., Ball C. A., Blake J. A. (2000). Gene ontology: tool for the unification of biology. *Nature Genetics*.

[19] Kanehisa M., Goto S. (2000). KEGG: kyoto encyclopedia of genes and genomes. *Nucleic Acids Research*.

[20] Sharma D., Surolia A., Dubitzky W., Wolkenhauer O., Cho K.-H. (2013). Degree Centrality. *Encyclopedia of Systems Biology, Dubitzky W*.

[21] Shannon P., Markiel A., Ozier O. (2003). Cytoscape: a software Environment for integrated models of biomolecular interaction networks. *Genome Research*.

[22] Tomczak K., Czerwińska P., Wiznerowicz M. (2015). The Cancer Genome Atlas (TCGA): An immeasurable source of knowledge. *Wspolczesna Onkologia*.

[23] Martin Bland J., Altman D. G. (1998). Survival probabilities (the Kaplan-Meier method). *British Medical Journal*.

[24] Alberti C., Timsit J.-F., Chevret S. (2005). Survival analysis: The log rank test. *Revue des Maladies Respiratoires*.

[25] Noerholm M., Balaj L., Limperg T. (2012). RNA expression patterns in serum microvesicles from patients with glioblastoma multiforme and controls. *BMC Cancer*.

[26] Sing T., Sander O., Beerenwinkel N., Lengauer T. (2005). ROCR: visualizing classifier performance in R. *Bioinformatics*.

[27] Long Fei, Su Jia-Hang, Liang Bin, Su Li-Li, Jiang Shu-Juan (2015). Identification of Gene Biomarkers for Distinguishing Small-Cell Lung Cancer from Non-Small-Cell Lung Cancer Using a Network-Based Approach. *BioMed Research International*.

[28] Zhang B., Horvath S. (2005). A general framework for weighted gene co-expression network analysis. *Statistical Applications in Genetics and Molecular Biology*.

[29] Ni Y., Zhang F., An M., Yin W., Gao Y. (2018). Early candidate biomarkers found from urine of glioblastoma multiforme rat before changes in MRI. *SCIENCE CHINA Life Sciences*.

[30] Dent P., Yacoub A., Park M., etal. (2008). Searching for a cure: gene therapy for glioblastoma. *Cancer Biology & Therapy*.

[31] Dmitrenko V. V., Bojko O. I., Shostak K. O. (2007). Characterization of genes, down-regulated in human glioma, potential tumor suppressor genes. *Biopolymers & Cell*.

[32] Sim H., Hu B., Viapiano M. S. (2009). Reduced expression of the hyaluronan and proteoglycan link proteins in malignant gliomas. *The Journal of Biological Chemistry*.

[33] Delic S., Thuy A., Schulze M. (2015). Systematic Investigation of CMTM Family Genes Suggests Relevance to Glioblastoma Pathogenesis and CMTM1 and CMTM3 as Priority Targets. *Genes, Chromosomes and Cancer*.

[34] Pruitt H. C., Devine D. J., Samant R. S. (2016). Roles of N-Myc and STAT interactor in cancer: From initiation to dissemination. *International Journal of Cancer*.

[35] Meng D., Li X., Zhang S. (2015). Genetic variants in N-myc (and STAT) interactor and susceptibility to glioma in a Chinese Han population. *Tumour Biology the Journal of the International Society for Oncodevelopmental Biology & Medicine*.

[36] Nomura H., Uzawa K., Ishigami T. (2008). Clinical significance of gelsolin-like actin-capping protein expression in oral carcinogenesis: An immunohistochemical study of premalignant and malignant lesions of the oral cavity. *BMC Cancer*.

[37] Yun D., Wang Y., Meng D. (2018). Actin-capping protein CapG is associated with prognosis, proliferation and metastasis in human glioma. *Oncology Reports*.

[38] Yang B.-Y., Song J.-W., Sun H.-Z. (2018). PSMB8 regulates glioma cell migration, proliferation, and apoptosis through modulating ERK1/2 and PI3K/AKT signaling pathways. *Biomedicine & Pharmacotherapy*.

[39] Arima K., Kinoshita A., Mishima H. (2011). Proteasome assembly defect due to a proteasome subunit beta type 8 (PSMB8) mutation causes the autoinflammatory disorder, Nakajo-Nishimura syndrome. *Proceedings of the National Acadamy of Sciences of the United States of America*.

[40] Carroll M. J., Nusblat L., Roth C. M. Role of inflammatory pathway and cells on glioma cell response to chemotherapy.

[41] Teng D.-C., Sun J., An Y.-Q. (2016). Role of PHLPP1 in inflammation response: Its loss contributes to gliomas development and progression. *International Immunopharmacology*.

[42] Li Y., Min W., Li M. (2016). Identification of hub genes and regulatory factors of glioblastoma multiforme subgroups by RNA-seq data analysis. *International Journal of Molecular Medicine*.

[43] Zhu T., Xie P., Gao Y. F. (2018). Nucleolar and spindle-associated protein 1 is a tumor grade correlated prognosis marker for glioma patients. *Cns Neuroscience & Therapeutics*.

[44] Tuo-Ye X. U., Zhen-Nan Y. U., Zhao P. L. (2016). Expression of NUSAP1 in glioma cells and its effects on LN229 cell. *Journal of Clinical Neurosurgery*.

[45] Karen S McColl A. E. (2014). Acidosis Sensing Receptor GPR65 Correlates with Anti-Apoptotic Bcl-2 Family Member Expression in CLL Cells: Potential Implications for the CLL Microenvironment. *Journal of Leukemia*.

[46] Ail D., Rüfenacht V., Caprara C., Samardzija M., Kast B., Grimm C. (2015). Increased expression of the proton-sensing G protein-coupled receptor Gpr65 during retinal degeneration. *Neuroscience*.

